# Dental Friendship

**Published:** 1908-11-15

**Authors:** C. P. Elder


### DENTAL FRIENDSHIP.

BY DR. C. P. ELDER.

While I *laud* old time friendships, cemented as they are by the memories of years, I believe that they, while perhaps our *chiefest*, should not be our *only* friendships among our professional brethren. For, in the various towns and cities wherein we take up our abode after our school days are over, and wherein we undertake the responsibilities of a more exacting life and a fuller citizenship there are usually others,—following the same pursuits, striving for the same ends, making the same successes, the same failures, as ourselves; urged onward by the same ideals, the same incentives, handicapped by the same odds and the same stubborn problems—in a word, our fellow dentists.

These, by their very profession, by their similar work, thoughts, desires, failures and successes, should be *ripest* for *our* friendship, and *we* for *theirs.* "Friendship is mutual;" who can so readily understand us and our viewpoint, our doubts, our fears, our cares and anxieties, our discouragements, our hopes, our plans and our accomplishments as our fellow craftsman? No one is as well fitted as he.

To many my words are needless, for your closest friends are not only in the dental profession, but are also your townsmen. To you I extend my *sincere* congratulations and felicitations that such is *your* good fortune, and *to* you I award my *heartiest* applause. You are *favored* of the gods. And to *you* who have allowed envy or malice or bickerings or misunderstandings to interfere with your friendly relations to your brethren, or have, through indifference, or lack of proper consideration of the matter, never formed such relations in your home town, I offer my *deepest sympathy*, and beg of you for your own sake, for your brother practitioner's sake and for the sake of the high calling in which you are engaged, to at once remedy your mistakes.

I realize, fully, the many causes of unfriendly and even openly hostile attitudes between members of our cult, located in the same town. But I claim nearly every one of them to be insufficient. If your *own* hands are clean, if *your* heart is warm toward "Dr. John Doe," if *you* desire, and make *known* your desire to have *his* friendship and good will, it cannot be that he will long withstand *you*. He, too, is human and desires friends. He may not understand you at first, he may be suspicious, worldly wisdom may have rendered him so; he may be diffident, or indifferent, yet his friendship, once won, should enrich your life and be well worth *any effort* at the outset.

I realize, also, the fact that there are *some* men with whom we may be thrown in contact, that are well nigh impossible as close, warm friends. Yet that they are in the *minority*, in our ranks, I am optimist enough to fully believe. And even with *such* we may be on good terms, rather than at crossed swords. Sincerely and honestly and consistently proffered *good will* and *friendliness* will make even a *scoundrel* a better man and *may* entirely reclaim him. The condition which is *most regretable* is that of two of our members, neighbors, each of whom has the mind, the heart, the qualifications generally, which are sufficient for the making of a good friend and companion, and who have been separated by mischance, accident or misunderstanding—or who have never formed a friendship because of doubts or suspicions or a lack of seeing the necessity of it. To men in *this unfortunate predicament*, do I most particularly address myself. Get together! You who are here, and know the value and delights of our association in this Society, when you go home, advance *more* than half way toward your brother (who does not come, perchance, but who *should* come) and start a "Dental Society" of two with him. We need not be of the same age, nor have the same likes and dislikes, we need not have the same opinions nor the same view-points, to be friendly and have fraternal intercourse. From the association with our *older* brother we can glean many a golden thought-grain as he speaks words of wisdom, based on his years of experience; and we, the younger man, can do *much* to free *his* labors from care by our cheerful thoughts, our buoyant hopes and our enthusiasm—because of our youth and expectations—even if we sometimes *pain* him by departing from the paths of procedure he advises, and he shakes his doubting head over our latest and newest "wild idea."—Abstract from October *Western Journal.*

